# Molecular epidemiology and antimicrobial susceptibility of *Clostridium difficile* isolates from two Korean hospitals

**DOI:** 10.1371/journal.pone.0174716

**Published:** 2017-03-29

**Authors:** Asiimwe Nicholas, Yu Kyung Kim, Won-Kil Lee, Gati Noble Selasi, Seok Hyeon Na, Hyo Il Kwon, Yoo Jeong Kim, Hae Sook Lee, Kyung Eun Song, Jeong Hwan Shin, Je Chul Lee

**Affiliations:** 1 Department of Microbiology, Kyungpook National University School of Medicine, Daegu, Republic of Korea; 2 Department of Laboratory Medicine, Yeungnam University College of Medicine, Daegu, Republic of Korea; 3 Department of Laboratory Medicine, Kyungpook National University School of Medicine, Daegu, Republic of Korea; 4 Department of Laboratory Medicine, Inje University College of Medicine, Busan, Republic of Korea; Cleveland Clinic, UNITED STATES

## Abstract

*Clostridium difficile* is one of the main etiological agents causing antibiotic-associated diarrhea. This study investigated the genetic diversity of 70 toxigenic *C*. *difficile* isolates from two Korean hospitals by employing toxinotyping, ribotyping, multilocus sequence typing (MLST), and pulsed-field gel electrophoresis (PFGE). Toxin gene amplification resulted in 68 A⁺B⁺ and two A^-^B^+^ isolates. Most isolates (95.7–100%) were susceptible to daptomycin, metronidazole, and vancomycin. Seventy *C*. *difficile* isolates were classified into five toxinotypes, 19 ribotypes, 16 sequence types (STs), and 33 arbitrary pulsotypes. All *C*. *difficile* isolates of ribotype 018 (*n* = 38) were classified into ST17, which was the most prevalent ST in both hospitals. However, *C*. *difficile* isolates of ST17 (ribotype 018) exhibited pulsotypes that differed by hospital. ST2 (ribotype 014/020), 8 (ribotypes 002), 17 (ribotype 018), and 35 (ribotypes 015) were detected in both hospitals, whereas other STs were unique to each hospital. Statistical comparison of the different typing methods revealed that ribotyping and PFGE were highly predictive of STs. In conclusion, our epidemiological study indicates that *C*. *difficile* infections in both hospitals are associated with the persistence of endemic clones coupled with the emergence of many unique clones. A combination of MLST with PFGE or ribotyping could be useful for monitoring epidemic *C*. *difficile* strains and the emergence of new clones in hospitals.

## Introduction

*Clostridium difficile*, a gram-positive obligate anaerobic spore-forming bacillus, is a leading etiological agent of antibiotic-associated clinical manifestations that range from mild diarrhea to serious pseudomembranous colitis. *C*. *difficile* infection (CDI) is of great concern because of its prevalence and high morbidity and mortality rates in both nosocomial and community-acquired infections worldwide [1, 2]. The toxins, enterotoxin A (TcdA) and cytotoxin B (TcdB), produced by toxigenic *C*. *difficile* strains play a crucial role in the pathogenesis of CDI. However, there are increasing reports of TcdA-negative/TcdB-positive (A^-^B^+^) variants in Korea and other countries [3, 4]. The prevalence of A^-^B^+^ or A^+^B^+^ *C*. *difficile* strains varies considerably across hospitals and countries [5, 6]. In addition, some *C*. *difficile* strains, either toxigenic or non-toxigenic, produce a multi-domain, actin ADP-ribosylating binary toxin (CDT), which is thought to modify actin in a manner that facilitates bacterial adhesion [7]. Outbreaks or epidemics have been attributed to specific types of *C*. *difficile*, such as restriction endonuclease analysis group BI, North America pulsed-field gel electrophoresis (PFGE) type 1, and polymerase chain reaction (PCR) ribotype 027 (NAP1/BI/027) and PCR ribotype 078 [8, 9]. PCR ribotypes 017, 014/020, and 018 have also caused nosocomial outbreaks worldwide [10–12].

Several epidemiological tools such as PCR ribotyping, toxinotyping, PFGE, and multilocus sequence typing (MLST) have been used to study genotypic and phenotypic variation in *C*. *difficile*. PCR ribotyping is the most commonly used typing technique, and this technique has the power to differentiate between *C*. *difficile* isolates originating from different hospitals and countries [5]. However, band-based typing tools such as PCR ribotyping and PFGE can be difficult to interpret and use in phylogenetic analyses of *C*. *difficile* isolates. In recent years, sequence-based typing tools with online support, such as MLST (http://pubmlst.org/cdifficile), have been established and widely used for global epidemiological study of *C*. *difficile* isolates.

Several epidemiological studies of CDI in Korea have been reported [12–14]; however, these studies focused on the identification of hypervirulent epidemic strains and examination of genetic diversity among *C*. *difficile* isolates using PCR ribotyping. Ribotypes 001, 017, and 014/020 of *C*. *difficile* were the most prevalent in Korea in the last decade, but their prevalence has changed through time, with ribotype 001 decreasing and ribotypes 017, 014/020, and 018 increasing [12–14]. A hypervirulent strain of ribotype 027 was also reported in a Korean hospital; however, few CDIs have been caused by this ribotype [14]. However, sequence types (STs) of *C*. *difficile* determined by MLST and their correlation with PCR ribotypes, pulsotypes established by PFGE, and toxinotypes have not yet been studied in Korea. This study investigated the clonal distribution of *C*. *difficile* isolates from two Korean hospitals using toxinotyping, ribotyping, PFGE, and MLST. In addition, the congruence of different epidemiological typing methods was assessed.

## Materials and methods

### Bacterial isolates and culture conditions

A total of non-duplicate toxigenic 70 *C*. *difficile* isolates from the diarrheal stools of patients who had visited or were admitted to two Korean hospitals, Kyungpook National University Hospital (KNUH) in Daegu (*n* = 31) and Busan Paik Hospital (BPH) in Busan (*n* = 39), between 2013 and 2015 were used in this study. Two hospitals were university-affiliated tertiary hospitals, and KNUH and BPH had a total of 904 and 895 beds, respectively. All *C*. *difficile* isolates were obtained from Kyungpook National University Hospital Culture Collection for Pathogens (KNUH-CCP). *C*. *difficile* isolates collected in the KNUH-CCP were from representative cases of CDIs based on prevalence of CDIs in each hospital, phenotypes of the isolates, and clinical information of the patients during the study periods. Species were identified by 16S ribosomal RNA gene sequence analysis. Two reference strains, ATCC 43255 from the American Type Culture Collection (Manassas, VA, USA) and KCTC 5009 from the Korea Collection for Type Cultures (Osong, Korea), were used. Bacterial culturing, management, and storage were performed based on protocols described by Edwards *et al* [15]. *C*. *difficile* isolates were anaerobically cultured at 37°C on Brain-Heart Infusion (BHI) medium (BD Biosciences, San Jose, CA, USA) supplemented with sodium taurocholate (10% w/v, Sigma-Aldrich, St. Louis, MO, USA), l-cysteine (10% w/v, Sigma-Aldrich), hemin in 1 M sodium hydroxide (10% v/v, Sigma-Aldrich), and vitamin K1 in 95% ethanol (0.02% v/v, Sigma-Aldrich).

### Genomic DNA extraction

Genomic DNA was isolated from two or three colonies of *C*. *difficile* grown on BHI medium overnight in anaerobic conditions. DNA was extracted using a DNA Extraction Kit (Biofact Co., Korea) according to the manufacturer’s instructions. The extracted DNA was used for MLST, toxinotyping, and PCR analysis of toxin genes.

### Amplification of toxin genes

PCR was performed to detect toxin genes of *C*. *difficile*. The primers sets used were NK11 and NK9 for the repetitive domain of the toxin A gene (*tcdA*), NK104 and NK105 for the toxin B gene (*tcdB*), cdtApos and cdtArev for the binary toxin A gene (*cdtA*), and cdtBpos and cdtBrev for the binary toxin B gene (*cdtB*) [16–18]. The absence of the pathogenicity locus (PaLoc) was confirmed using the primers Lok1 (5′-AAA ATA TAC TGC ACA TCT GTA TAC-3′) and Lok3 (5′-TTT ACC AGA AAA AGT AGC TTT AA-3′) as described by Rupnik (http://www.mf.uni-mb.si/tox/). PCR products were electrophoresed on 1% agarose gel and bands were visualized using a ChemiDoc MP System (Bio-Rad, Hercules, CA, USA).

### PFGE

PFGE was performed according to previously described protocol with some modifications [19]. Briefly, bacteria were cultured in BHI broth until the OD_600_ reached 1.8–2.0. The bacterial cells were lysed with 100 μl of 2× lysis buffer (2 M NaCl_2_, 200 mM EDTA (pH 8.0), 1% Brij-58, 0.4% sodium deoxycholate, 1% sodium lauryl sarcosine, lysozyme 4 mg/ml, RNase 100 mg/ml, and mutanolysin 40 U/ml). Plugs containing the lysed bacterial cells were prepared using 2% PFGE-grade low melting point agarose. The plugs were then incubated in 1× lysis buffer over night at 37°C, followed by incubation with TE buffer (10 mM Tris-HCl, 1 mM EDTA) containing 10 mg/ml proteinase K, for 24 h at 50°C, with buffer changes. The plugs were then washed with TE buffer (10 mM Tris-HCl, 1 mM EDTA) and digested with 50 U of *Sma*I (Thermo Fisher Scientific, Waltham, MA, USA) for 6 h at 25°C. The digested products were electrophoresed on a 1% PFGE-grade agarose gel using a CHEF Mapper system (Bio-Rad). The band patterns were analyzed with GelCompar II software (Applied Maths, Kortijk, Belgium) to produce a dendrogram. A dendrogram was constructed using the unweighted pair-group method with arithmetic mean clustering. Pulsotypes were arbitrarily classified based on a similarity value of 0.85.

### PCR ribotyping

PCR ribotyping of *C*. *difficile* isolates was performed as previously described [20]. PCR amplification was performed using the primers 5′-CTG GGG TGA AGT CGT AAC AAG G-3′ and 5′-GCG CCC TTT GTA GCT TGA CC-3′. The PCR conditions comprised an initial denaturation step at 95°C for 3 min, 30 cycles of denaturation at 95°C for 30 S, annealing at 55°C for 40 S, and extension at 72°C for 70 S, and a final elongation step at 72°C for 5 min. Amplification products were electrophoresed on 2% agarose gels. The electrophoretic patterns were compared with those available in a published library [21]. Nomenclature of new ribotypes that did not match with any UK ribotypes was based on the Kim’s ribotyping scheme [22].

### Toxinotyping

The 19-kb PaLoc region comprises toxin genes (*tcd*A and *tcd*B) and additional open reading frames (*tcd*C, *tcdD*, and *tcdE*) [23]. Toxinotyping is a restriction fragment length polymorphism typing method used to differentiate *C*. *difficile* isolates based on sequence distinctions in the PaLoc region [24]. Toxinotyping was conducted on both toxigenic and non-toxigenic *C*. *difficile* isolates as described by Rupnik (http://www.mf.uni-mb.si/tox/). PCR products and their restriction fragments were analyzed on 1% agarose gels. Toxinotypes of *C*. *difficile* isolates were determined by comparing their band patterns with that of the standard strain ATCC 43255. Patterns that visually differed from the pattern of this strain were compared with other known toxinotypes.

### MLST

*C*. *difficile* isolates were subjected to MLST to identify allelic polymorphisms at seven loci (*adk*, *atp*A, *dxr*, *gly*A, *rec*A, *sodA*, and *tpi*) as previously described by Griffiths *et al*. [25]. Isolates were assigned to STs and clades in the *C*. *difficile* MLST database (http://pubmlst.org/cdifficile). Clonal relationships among STs, based on allelic polymorphisms, were determined using the EBURST program (eBURST version 3 (http://eburst.mlst.net/), as previously described [26].

### Antimicrobial susceptibility testing

The minimum inhibitory concentrations (MICs) of ciprofloxacin (CIP), daptomycin (DAP), linezolid (LNZ), metronidazole (MTZ), moxifloxacin (MXF), rifampicin (RIF), and vancomycin (VAN) were determined by E-test according to the manufacturer’s instructions (bioMérieux, Marcy l'Etoile, France). *Bacteroides fragilis* ATCC 25285 was used for quality control. Interpretation of antimicrobial susceptibility was based on the guidelines of the Clinical Laboratory Standards Institute (CLSI) [27] and European Committee on Antimicrobial Susceptibility Testing (EUCAST) [28].

### Comparison of the typing methods

The Simpson’s index of diversity (SID), which measures the probability that two subjects randomly selected from a sample population belong to dissimilar types, was used as a discriminatory index. The adjusted Wallace coefficient (WC) was used to evaluate the bidirectional agreement between typing methods [29]. The SID and adjusted Wallace coefficient were calculated using a web-based tool (http://www.comparingpartitions.info/).

## Results

### Isolation of *C*. *difficile*

Seventy *C*. *difficile* isolates were obtained from patients who diagnosed CDIs. Patients were admitted to the internal medicine (*n* = 37), emergency room (*n* = 12), neurosurgery (*n* = 6), general surgery (*n* = 3), neurology (*n* = 4), outpatient (*n* = 2), pediatric (*n* = 2), rehabilitation (*n* = 2), orthopedic surgery (*n* = 1), and urology (*n* = 1) wards. Fifty isolates were from patients over 60 years of age, 14 were from patients aged 30–59 years, and six were from patients aged 10–30 years.

### Toxin gene profiles

**Fig 1:**
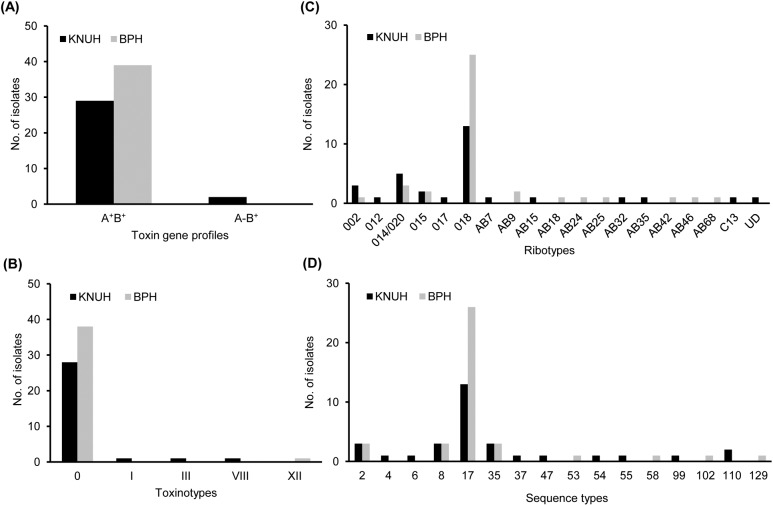
**Distribution of toxin gene profiles (A), toxinotypes (B), PCR ribotypes (C), and sequence types (D) among 70 *C*. *difficile* isolates from two Korean hospitals.** In Fig 1C, UD means that the isolate was not assigned to any known ribotype. KNUH, Kyungpook National University Hospital; BPH, Busan Paik Hospital.

Sixty-eight isolates were A^+^B^+^ and two were A^-^B^+^ (Fig 1A). The *cdt* gene encoding the binary toxin was identified in one isolate (CDN7725), and toxin profile of this isolate was A^+^B^+^CDT^+^. Two A^-^B^+^-type *C*. *difficile* isolates were detected in KNUH.

### Toxinotypes

Sixty-six isolates exhibited band patterns identical to that of the standard strain ATCC 43255; they were assigned to toxinotype 0 (Fig 1B). Four isolates were found to carry a whole or part of PaLoc and were classified into toxinotypes I, III, VIII, and XII. *C*. *difficile* isolates of toxinotypes I, III, and VIII were from the internal medicine ward in KNUH, whereas the single toxinotype XII isolate was from the emergency room in BPH.

### PCR ribotypes

Sixty-nine *C*. *difficile* isolates were categorized into 18 different PCR ribotypes (Fig 1C). One *C*. *difficile* isolate from KNUH could not be assigned to any known ribotype. PCR ribotype 018 was predominant, accounting for 38 isolates (25 from BPH and 13 from KNUH), followed by ribotypes 014/020 (*n* = 8), 015 (*n* = 4), 002 (*n* = 4), and AB9 (*n* = 2). The remaining 14 *C*. *difficile* isolates represented 14 different ribotypes. PCR ribotypes 018, 014/020, 015, and 002 were detected in both hospitals, whereas other ribotypes were unique to each hospital.

### STs

The 70 *C*. *difficile* isolates were classified into 16 STs (Fig 1D). ST17 was the most prevalent (*n* = 39), followed by ST8 (*n* = 6), ST35 (*n* = 6), ST2 (*n* = 6), and ST110 (*n* = 2). The remaining 11 STs were represented by single isolates. Twelve and eight STs were identified in KNUH and BPH, respectively. Sixty-eight A^+^B^+^ were classified into 18 ribotypes and 15 STs. Fourteen isolates from emergency room and outpatients belonged to five ribotypes and STs, respectively. With the exception of ribotype AB68, all *C*. *difficile* isolates of ribotype 018 were classified into ST17. Based on allelic polymorphisms, STs were allocated to clonal complexes using the eBURST program (http://eburst.mlst.net/). Of the 16 STs, eight STs were placed into three different groups; group 1 [ST2 (founder), ST102 (single locus variant [slv] of ST2 in *sodA*), and ST110 (slv of ST2 in *rec*A)], group 2 [ST4 (founder), ST17 (slv of ST4 in *tpi*), and ST53 (slv of ST4 in *atp*A)], and group 3 [ST99 (slv of ST55 in *adk*) and ST55]. Group 3 had no founding genotype. The remaining eight STs were singletons.

### PFGE analysis

**Fig 2:**
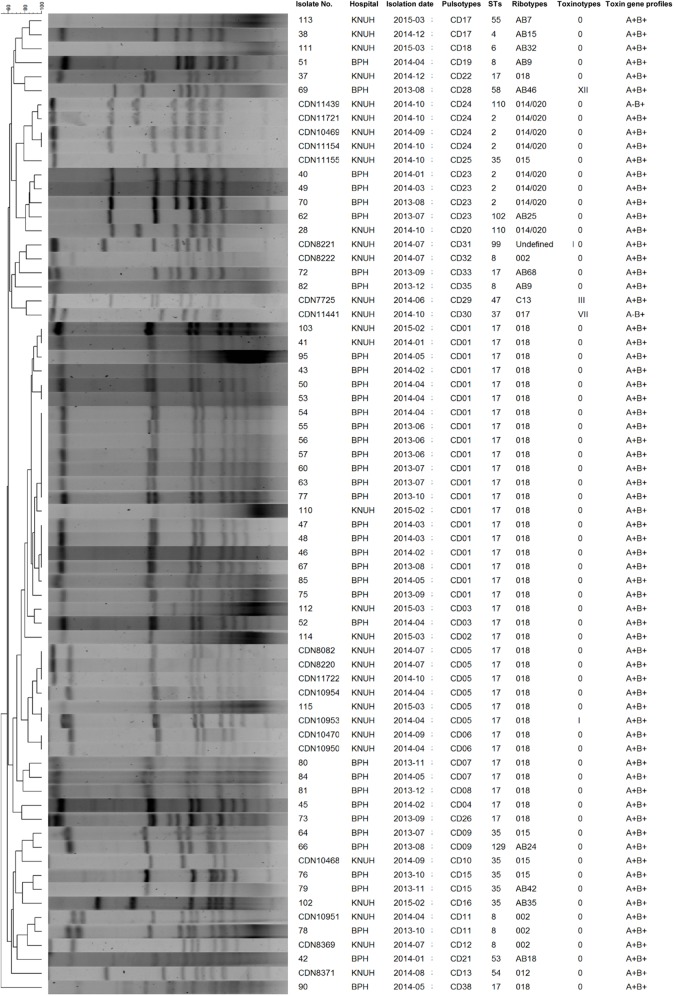
Dendrogram showing 33 pulsotypes obtained by pulsed-field gel electrophoresis of 70 *C*. *difficile* isolates. Genomic DNA was digested with *Sma*I. The unweighted pair group method with arithmetic mean clustering method was used with the Dice coefficient with position tolerance and optimization of 1.2%. Pulsotypes were arbitrarily assigned based on a similarity value of 0.85. Undefined, isolates that were not assigned to any known ribotype; AB and C, ribotype nomenclature adopted by Kim’s ribotyping scheme [3, 22].

All 70 *C*. *difficile* isolates were classified into 33 arbitrary pulsotypes based on a similarity value of 0.85 (Fig 2). Arbitrary pulsotype 1 (CD01) was the most prevalent, accounting for 20 isolates, followed by pulsotype CD05 (*n* = 6). *C*. *difficile* isolates showing two prevalent pulsotypes, CD01 and CD05, belonged to ribotype 018 and ST17. The 39 *C*. *difficile* isolates from BPH yielded 16 pulsotypes, with pulsotype CD01 predominant (*n* = 18), whereas the 31 *C*. *difficile* isolates from KNUH produced 20 pulsotypes, with pulsotype CD05, belonging to ribotype 018, the most prevalent (*n* = 6). The binary toxin gene-positive isolate CDN7725 belonged to ribotype C13, ST47, toxinotype III, and pulsotype CD29.

### Antimicrobial resistance

The distribution of MICs of antimicrobial agents against *C*. *difficile* isolates is shown in Table 1. Antimicrobial resistance rates of the 70 *C*. *difficile* isolates were as follows: MXF, 55.7%; RIF, 27.1%; DAP, 4.3%; and MTZ, 2.9%. No isolates were resistant to VAN. Sixty-seven isolates showed very high CIP MICs (≥ 32 μg/ml). One *C*. *difficile* isolate (CDN10470) with ST17 and ribotype 018 was resistant to CIP, DAP, MTZ, MXF, and RIF. Two ST17 isolates were resistant to MTZ. Of the 39 *C*. *difficile* isolates resistant to MXF, 32 belonged to ST17. There was no significant difference in antimicrobial resistance rates of *C*. *difficile* isolates between hospitals.

**Table 1:** Antimicrobial susceptibility of 70 *C*. *difficile* isolates from two Korean hospitals.

Antimicrobial agents	MIC (μg/ml) (No. of isolates)	% Resistance
Ciprofloxacin	0.125 (1), 0.25 (1), 1 (1), > 32 (67)	NA^a^
Daptomycin	0.125 (1), 0.25 (4), 0.38 (4), 0.5 (19), 0.75 (12), 1 (24), 1.5 (3), 4 (1), 12 (2)	4.3^b^
Linezolid	0.5 (1), 0.75 (6), 1.0 (8), 1.5 (14), 2 (29), 3 (12)	NA
Metronidazole	0.002 (3), 0.016 (1), 0.032 (1), 0.064 (5), 0.094 (11), 0.125 (12), 0.19 (13), 0.25 (7), 0.38 (8), 0.5 (6), 2 (1), > 32 (2)	2.9^c^
Moxifloxacin	0.064 (1), 0.38 (2), 0.5 (1), 0.75 (2), 1 (9), 1.5 (12), 2 (3), 4 (1), > 32 (39)	55.7^c^
Rifampicin	0.002 (45), 0.003 (6), 0.004 (3), 0.006 (4), 0.008 (1), 0.023 (1), 0.032 (1), 0.047 (1), 0.064 (1), 8 (1), > 32 (6)	27.1^b^
Vancomycin	0.016 (1), 0.125 (5), 0.19 (2), 0.25 (8), 0.38 (6), 0.5 (35), 0.75 (11), 1 (2)	0^b^

^a^ NA, MIC interpretive breakpoints have not been determined by CLSI or EUCAST.

^b^ Based on EUCAST epidemiological cut-off values (daptomycin, 4 μg/ml; rifampicin 0.004 μg/ml; vancomycin, 2 μg/ml) [28].

^c^ Based on resistance criteria by CLSI (metronidazole, ≥ 32 μg/ml; moxifloxacin, ≥ 8 μg/ml) [27].

### Statistical comparison of the performance of the epidemiological typing tools

SID was used to assess the ability of each epidemiological tool to differentiate between two test samples picked randomly from the sample population. Of the typing methods used in this study, PFGE, which yielded 33 arbitrary pulsotypes, exhibited the highest SID of 0.907, whereas toxinotyping, which defined five different types, showed the lowest SID of 0.112 (Table 2). The WC was used to analyze the degree of concordance between typing schemes (Table 3). Ribotyping’s congruence with MLST was greatest, with a WC of 0.976 (95% CI, 0.957–0.995), and PFGE showed agreement with MLST, with WC of 0.961 (95% CI, 0.938–0.985). However, low congruence was observed between toxinotyping and PFGE (WC = 0.010; 95% CI, 0.000–0.079) and toxinotyping and ribotyping (WC = 0.031; 95% CI, 0.000–0.211).

**Table 2:** Comparison of four different epidemiological typing methods using Simpson's index of diversity.

Typing method^a^	Number of partitions	Simpson's index of diversity (95% CI)
PFGE	33	0.907 (0.852–0.963)
Ribotyping	19	0.692 (0.574–0.810)
MLST	16	0.674 (0.555–0.793)
Toxinotyping	5	0.112 (0.009–0.215)

^a^ PFGE, pulsed-field gel electrophoresis; MLST, multilocus sequence typing.

**Table 3:** Concordance values of four different typing methods estimated using the adjusted Wallace coefficient.

Typing method^a^	PFGE	Ribotyping	MLST	Toxinotyping
PFGE	-	0.961 (0.938–0.985)	0.947 (0.916–0.978)	0.800 (0.543–1.000)
Ribotyping	0.221 (0.065–0.377)^b^	-	0.976 (0.957–0.995)	0.555 (0.000–1.000)
MLST	0.200 (0.050–0.351)	0.899 (0.767–1.000)	-	0.568 (0.000–1.000)
Toxinotyping	0.010 (0.000–0.079)	0.031 (0.000–0.211)	0.035 (0.000–0.221)	-

^a^ PFGE, pulsed-field gel electrophoresis; MLST, multilocus sequence typing.

^b^ The values indicate 95% confidence intervals.

## Discussion

This study reports, for the first time, the distribution of STs among *C*. *difficile* isolates from Korean hospitals. In addition, toxin gene profiling, toxinotyping, PFGE, and ribotyping were performed to explore the epidemiological characteristics of *C*. *difficile* isolates from two Korean hospitals. Not only were international epidemic *C*. *difficile* clones, including ribotypes 018 (ST17), 014/020 (ST2/ST110), and 015 (ST26/ST35) found in both study hospitals, but diverse sporadic clones unique to each hospital were also detected.

Toxigenic *C*. *difficile* strains secrete one or both TcdA and TcdB toxins. A^-^B^+^ *C*. *difficile* variants cannot secrete TcdA owing to deletions in the repetitive sequence of the *tcdA* gene. This toxin variant strain was first described in the early 1990s [30, 31]. A^-^B^+^ variants were found to constitute less than 10% of the toxigenic isolates from European countries and Japan, but were detected in up to 40% of isolates in Korea and Thailand [3, 10–12]. In this study, two A^-^B^+^ *C*. *difficile* isolates were identified only in KNUH. Earlier studies demonstrated a high correlation between toxin types and STs; A^-^B^+^ variants from China and Thailand were found to belong to ST37 and ST45 (ribotype 017), respectively. Moreover, A^-^B^+^ variants from Korea belonged to ribotype 017 [22]. However, two A^-^B^+^ variants tested in this study represented ribotypes 014/020 (ST110) and 017 (ST37), respectively.

PCR ribotyping of *C*. *difficile* isolates revealed that ribotype 018 (54.3%) was the most prevalent in both hospitals. Our results were consistent with those of a previous study conducted in a single hospital in Korea, which also reported a high prevalence of ribotype 018 [14]. Another report showed that a ribotype from Japan, smz, which corresponds to ribotype 018, was a major clone occurring in both outbreak and non-outbreak CDIs [32]. Ribotype 018 is reported to be one of the most prevalent strains causing complicated infections in France and Italy [33–35]. The prevalence and clinical relevance of ribotype 018 can be attributed to the fact that strains belonging to this genotype are relatively resistant to fluoroquinolones and have a unique variant of the *splA* gene, which is speculated to confer a high degree of adhesiveness that contributes to the success of this genotype [36]. In this study, the resistance rate of ribotype 018 *C*. *difficile* isolates to MXF was 84.2% (32 of 38 isolates). Moreover, all *C*. *difficile* isolates of ribotype 018, except for one isolate, exhibited the highest CIP MICs (> 32 μg/ml). Ribotypes 017, 014/020, 015, 002, 001, and 012 are increasingly becoming potential epidemic strains. These ribotypes were prevalent among *C*. *difficile* isolates obtained over the past decade in Asian as well as European countries [5, 14, 32, 37], suggesting that they are globally circulating strains. Ribotypes 027, 014/020, UM11, 053/163, 002, 001, 078–126, 017, and 014 were the chief agents in outbreaks and epidemics in North American countries [38–41]. In an Australian survey of the hospital-acquired and community-acquired CDIs, ribotypes 014/020, 002, 054, 056, and 070 were the most prevalent in 2010, and *C*. *difficile* isolates of ribotypes 014/020, 056, 002, and 018 were the prevalent between 2012 and 2014 [42, 43]. Regarding Asian countries, the most common ribotypes in China have been 017, 012, and 046 [44], whereas ribotypes 018, 001, 017, and 014/020 are prevalent in Japan and Korea [11, 14]. We also found that most of the ribotypes were unique to a given hospital, with four ribotypes (AB7, AB15, AB32, and AB35) found in KNUH and seven ribotypes (AB9, AB18, AB24, AB25, AB42, AB46, and AB68) found in BPH. Fourteen *C*. *difficile* isolates from community-acquired CDIs, two from outpatients and 12 from emergency room, were analyzed. Two isolates from outpatients were A^+^B^+^ and ribotype 018. Twelve A^+^B^+^ isolates from emergency room showed five different ribotypes, 018 (*n* = 6), 014/020 (*n* = 3), AB15 (*n* = 1), AB35 (*n* = 1), and AB46 (*n* = 1). These results suggest that there is no difference in major clones of *C*. *difficile* between community-acquired and hospital-acquired CDIs. Our ribotyping results suggest that not only are epidemic *C*. *difficile* clones such as 018, 014/020, and 015 circulating in the study hospitals, but also that many sporadic clones are being introduced.

MLST of the 70 *C*. *difficile* isolates generated 16 different STs. We determined that ST17 (ribotype 018) was predominant, accounting for 38 isolates. One isolate of ribotype AB68 and pulsotype CD33 was found to belong to ST17. The other frequent types were ST2 (*n* = 6), ST8 (*n* = 6), and ST35 (*n* = 6). Eleven STs were represented by single isolates. MLST yielded a diverse pool of allelic polymorphisms, ranging from 4–10 per housekeeping gene locus. The *adk* gene, with four distinctive alleles, was the most conserved among the seven loci, whereas the *recA* gene had the highest diversity, exhibiting a total of 10 alleles. There was a high correlation between STs and ribotypes. For example, ST2, ST17, ST8, and ST35 correlated with the ribotypes 014/020 (100%), 018 (97.4%), 002 (66.7%), and 015 (66.7%), respectively. On the other hand, ST2 and ST110, which differed in the *recA* gene, belonged to the same ribotype, 014/020.

PFGE analysis of *C*. *difficile* isolates showed the highest discriminatory power of the epidemiological tools tested, with an SID of 0.907. Of the 33 arbitrary pulsotypes, CD01 (*n* = 20) and CD05 (*n* = 6) were the most prevalent; these pulsotypes correlated with ribotype 018 and ST17. Interestingly, 18 of the 20 CD01 isolates and all six CD05 isolates were from BPH and KNUH, respectively. ST2 was highly correlated with respective pulsotypes. However, the remaining 13 ST17 isolates were divided into 10 arbitrary pulsotypes. Moreover, ST8 and ST35 isolates showed diverse PFGE patterns and ribotypes. These results suggest that PFGE analysis is more valuable for intra-hospital epidemiological studies than other typing methods.

A statistical comparison of the different typing methods using the WC revealed that PCR ribotyping and PFGE results were highly predictive of MLST results; in other words, most isolates belonging to one ribotype or pulsotype grouped together in a single ST. However, ribotyping and MLST did not always yield similar outcomes. Occasionally, multiple STs were observed in a given ribotype [25, 45]. Conversely, multiple ribotypes clustered in a single ST. This observation suggests that no single epidemiological typing scheme can perfectly predict the outcome of the other typing methods. PFGE analysis yielded more groupings than any of the other three methods tested. However, a low correlation was found between the results of PFGE and these other methods. These results suggest that a combination of band-based typing (PFGE or PCR ribotyping) and sequence-based typing (MLST) could be a powerful tool for epidemiological study of *C*. *difficile* isolates.

This study has several limitations. First, we tested limited number of isolates from two hospitals. *C*. *difficile* isolates collected in our culture collection system for pathogens were from representative cases of CDIs, but it was possible that clonal diversity of *C*. *difficile* isolates is underestimated. However, in this study, 70 *C*. *difficile* isolates from two hospitals were classified into 19 ribotypes, 16 STs, and 33 arbitrary pulsotypes. Second, we did not analyze incidence of CDIs and presence of outbreaks in the study hospitals and clinical characteristics of the patients with CDIs regarding risk factors, severity of CDIs, and association with antibiotic exposure. These clinical parameters may influence the clonal distribution of *C*. *difficile* in the hospitals. However, despite these limitations, our study provides important epidemiological information of *C*. *difficile* isolates from two Korean hospitals concerning the persistence of specific international clones and the emergence of sporadic clones.

This study found that *C*. *difficile* strains of ST17/ ribotype 018 were the most prevalent in two Korean hospitals. No hypervirulent 027 strain was detected in this study. Interestingly, *C*. *difficile* strains of the epidemic ST17/ribotype 018 exhibited different pulsotypes in each hospital, with arbitrary pulsotype CD01 in BPH and CD05 in KNUH. With the persistence of epidemic clones and the emergence of sporadic clones, CDIs are becoming one of the important nosocomial infections in Korean hospitals. A combination of MLST with PFGE or PCR ribotyping could be useful for monitoring epidemic *C*. *difficile* strains and the emergence of new clones in hospitals.
